# Acylphloroglucinol Derivatives from the South African *Helichrysum niveum* and Their Biological Activities

**DOI:** 10.3390/molecules200917309

**Published:** 2015-09-18

**Authors:** Olugbenga K. Popoola, Jeanine L. Marnewick, Fanie Rautenbach, Emmanuel I. Iwuoha, Ahmed A. Hussein

**Affiliations:** 1 Chemistry Department, University of Western Cape, Private Bag X17, Bellville 7535, South Africa; E-Mails: 3318925@myuwc.ac.za (O.K.P.); eiwuoha@uwc.ac.za (E.I.I.); 2 Oxidative Stress Research Centre, Faculty of Health and Wellness Sciences, Cape Peninsula University of Technology, P. O. BOX 1906, Bellville 7535, South Africa; E-Mails: MarnewickJ@cput.ac.za (J.L.M.); rautenbachf@cput.ac.za (F.R.)

**Keywords:** *Helichrysum niveum*, asteraceae, phloroglucinols, antioxidant, anti-aging

## Abstract

Phytochemical investigation of aerial parts of *Helichrysum niveum* (*H. niveum*) using different chromatographic methods including semi-preparative HPLC afforded three new (**1**–**3**) and six known (**4**–**10**) acylphloroglucinols alongside a known dialcohol triterpene (**11**). The structures of the isolated compounds were characterized accordingly as 1-benzoyl-3 (3-methylbut-2-enylacetate)-phloroglucinol (helinivene A, **1**), 1-benzoyl-3 (2*S*-hydroxyl-3-methylbut-3-enyl)-phloroglucinol (helinivene B, **2**), 8-(2-methylpropanone)-3*S*,5,7-trihydroxyl-2,2-dimethoxychromane (helinivene C, **3**), 1-(2-methylbutanone)-4-*O*-prenyl-phloroglucinol (**4**), 1-(2-methylpropanone)-4-*O*-prennyl-phloroglucinol (**5**), 1-(butanone)-3-prenyl-phloroglucinol (**6**), 1-(2-methylbutanone)-3-prenyl-phloroglucinol (**7**), 1-butanone-3-(3-methylbut-2-enylacetate)-phloroglucinol (**8**), 1-(2-methylpropanone)-3-prenylphloroglucinol (**9**), caespitate (**10**), and 3β-24-dihydroxyterexer-14-ene (**11**). Excellent total antioxidant capacities were demonstrated by helinivenes A and B (**1** and **2**) when measured as oxygen radicals absorbance capacity (ORAC), ferric-ion reducing antioxidant power (FRAP), trolox equivalent absorbance capacity (TEAC) and including the inhibition of Fe^2+^-induced lipid peroxidation (IC_50_ = 5.12 ± 0.90; 3.55 ± 1.92) µg/mL, while anti-tyrosinase activity at IC_50_ = 35.63 ± 4.67 and 26.72 ± 5.05 µg/mL were also observed for **1** and **2**, respectively. This is the first chemical and *in vitro* biological study on *H. niveum*. These findings underpin new perspectives for the exploitation of these natural phenolic compounds in applications such as in the natural cosmeceutical and pharmaceutical sectors.

## 1. Introduction

The systemic process of aging in humans results in an imbalance between synthesis and degradation of the extracellular matrix. Overproduction of degradative enzymes and oxygen free radicals during chronological and photo-induced aging leads to degradation of the network and elastic skin collagen and hyper-synthesis of melanin. It is a complex process with multiple underlying influences including the probable involvement of inheritable and various environmental factors [1] that represents a major burden to the health care system. Aging is attributed to, but not limited to, the excessive accumulation of free radicals and other forms of reactive oxygen species (ROS), which are primarily generated in the body as a result of physiological and biochemical processes [2]. Other notable ways of accumulating ROS in the body, is through continuous body contact with a series of environmental cues (such as ultra-violet radiation and pollution), and lifestyle choices including (but not limited to) diet, smoking, status of concurrent diseases (e.g. diabetes), exercise and alcohol consumption [3]. Free radical accumulation, when above threshold level in the body, can cause cellular oxidative damage to important macromolecules such as proteins, lipids and deoxy nucleic acid (DNA), eventually leading to many chronic diseases such as cancer, aging and neurodegenerative disorders [4], and other degenerative diseases in humans [2]. The biologically relevant consequences of ultra-violet-A (UVA) exposure therefore include mainly photo-ageing and photocarcinogenesis [5].

*Helichrysum niveum* (L.) Less. (*H. niveum*) (synonyms; *Gnaphalium niveum* L., *Helichrysum ericifolium* Less. *var. metalasioides* (DC.) Harv., *Helichrysum metalasioides* (DC.) is an indigenous plant widely distributed along the Western Cape coast of South Africa, often on dunes and sandy soil. It has distinguishable acute white bracts, which is where its name *niveum*, or “snow white” plant [6] originated. From SciFinder and the dictionary of natural products database, no reports on scientific or ethno-medicinal value have been recorded. Our proposition was based on the chemotaxonomic relationship, which may possibly exist between *H. niveum* and other *Helichrysum* species. Previous findings expanded the knowledge about the phenolic profile of *Helichrysum* to be richly blessed with a large proportion of phloroglucinol derivatives [7,8,9,10,11,12] and terpenoids [13] among others. Dimeric phloroglucinols including arzanol with notable antioxidant, anti-inflammatory and antibacterial activities [13,14,15], has been documented in several *Helichrysum* species.

Following the ongoing exploration of untapped South African *Helichrysum* species embarked upon by our research group, we had previously documented scientific data on the biological application of South African *Helichrysum teretifolium* constituents as modulators of oxidative stress and skin aging [16]. The preliminary screening of the total extract of *H*. *niveum* showed very strong anti-oxidative capacity and an interesting chemical profile in the TLC analysis. Accordingly, we prioritized this plant extract and submitted it to phytochemical studies. The objective of this work was also directed to investigate the secondary metabolites of a methanol extract of *H. niveum* with a particular focus on the phenolic compounds and investigate them for possible total antioxidant capacity, as well as the tyrosinase, elastase and cholinesterase inhibitory activities.

## 2. Results and Discussion

The presence of terpenes and high amount of phenolics was first recognized by preliminary thin layer chromatographic screening of a methanol extract. Ten phloroglucinols derivatives (**1**–**10**) and a triterpene (**11**) were isolated from *H. niveum*. Their chemical structures were elucidated by extensive analyses of spectroscopic data (Table 1 and Table 2) as well as correlations with published data on literature. Three of the isolated compounds, helinivenes A–C (**1**–**3**) were reported for the first time. The total antioxidant capacities measured as FRAP, TEAC, ORAC and the inhibition of Fe (II)-induced microsomal lipid peroxidation ability of the total extract and its constituents are presented in Table 3, while the inhibition of aging-related enzymes measured using mushroom tyrosinase, elastase from pancreas porcine and acetylcholinesterase are presented in Table 4.

**Figure 1:**
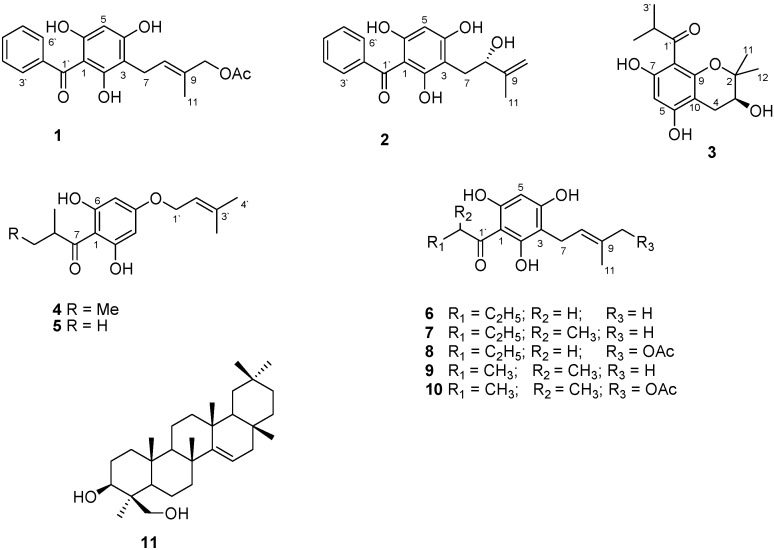
Chemical structures of compounds **1**–**11** isolated from *H. niveum*.

Compound **1** was obtained as yellow amorphous powder with UV (MeOH) spectrum showed λ_max_ at 300 nm. IR (KBr) spectrum showed absorption bands due to hydroxyl (3300 cm^−1^), carbonyl (1800 cm^−1^), terminal C=C (1680 cm^−1^), and aromatic (1410 cm^−1^). Its molecular formula was determined as C_20_H_20_O_5_. NMR data (Table 1 and Table 2) showed signals corresponding to di-substituted phloroglucinol at δ_H_ 6.07 s; δ_C_ 104.5, 163.4, 106.4, 163.2, 160.0 (C/each), 96.0 (CH), benzoyl group at δ_H_ 7.59–7.38 (5H); δ_C_ 200.3 (CO), 143.0 (C), 128.4 (X 2), 129.1 (X 2), 131.6, (CH/each) and a prenyl group with oxygenated and acetylated methyl terminal at δ_H_ 1.71 (CH_3_ δ_C_ 21.6), 3.38 (CH_2_, d, 7.5 Hz, δ_C_ 21.9), 4.82 (CH_2_, s, δ_C_ 63.7), 5.54 (CH, t, 7.5 Hz, δ_C_ 129.2(CH) and 130.4 (C). The ^1^H-NMR of **1** is very similar with 1-benzoyl-3-prenyl phloroglucinol [17]. The only difference observed is the presence of a signal at 4.82 (2H, s) and 2.02 of an acetyl group, both signals indicated the oxidation of a methyl group and acetylation and confirmed the presence of 3-methyl-2-butenylacetate side chain. HMBC confirmed the structure of the side chain and showed correlations between H-8/C-10, C-11, C-7, C-3 and H-11/C-9, C-10, C-8 and H-10/C-11, C-9, C-8 and CO (of the carbonyl group). Other 2D-NMR spectra supported the structure of **1** as 1-benzoyl-3(3-methylbut-2-enylacetate)-phloroglucinol, and given the trivial name helinivene A.

Compound **2** was obtained as a yellow amorphous powder. The UV spectrum of **2**, showed λ_max_ at 220, 280 nm. Its IR (KBr) spectrum showed bands of a hydroxyl group (3300 cm^−1^), terminal C=C (3100 cm^−1^), carbonyl (1800 cm^−1^), and aromatic (1410 cm^−1^). HRMS indicated the molecular formula of C_18_H_18_O_4_ with [M + 1]^+^ at 299.1220. The NMR of **2** is very similar to **1** except for the presence of signals of terminal olefinic methylene group at δ_H_ 4.97 s, 4.79 s (δ_C_ 110.8, 148.8), and oxygenated methine (δ_H_ 4.34 br d, *J* = 7.8 Hz; δ_C_ 77.7) and the absence of signals of H-8 (olefinic proton) and H-10 acetylated methylene group (Table 1 and Table 2). The above data indicated the presence of 2-hydroxyl-3-methylbut-3-enyl side chain. The HMBC correlations of **2** supported the structure as depicted in Figure 1 and showed correlations between H-11/C-9, C-10, C-8; H-8/C-10, C-11, C-3 and H-7/C-3, C-4, C-8, C-9 (among others). Direct correlation of optical rotation value (${\text{[α]}}_{D}^{20}$ −95, *c* 0.1 [reported ${\text{[α]}}_{D}^{20}$ −106 (*c* 0.1)] suggested *S* configuration of C-8 [18]. The forgoing data established the chemical structure of **2** as 1-benzoyl-3(2*S*-hydroxyl-3-methylbut-3-enyl)-phloroglucinol (helinivene B).

Compound **3** was obtained as a pale amorphous powder with similar spectroscopic data of phloroglucinol derivatives like **1** and **2**. It showed UV (MeOH) λ_max_ 314 nm, while IR (KBr) spectrum showed bands of a hydroxyl group (3300 cm^−1^), and carbonyl (1629 cm^−1^). The molecular formula of **3** was determined to be C_15_H_20_O_5_, which require six degree of unsaturation [*m*/*z* 279.1596]. The NMR data showed signals of 1-methyl-1-propanone side chain (Table 1 and Table 2), an aromatic proton at 5.99 s, and two oxygenated carbons at 80.0, 69.2, one of them showed a proton at δ_H_ 3.81, cyclic methylene group at δ_H_ 2.87, 2.51. The conjugated carbonyl and the aromatic ring accounted for five of the six observed degrees of unsaturation, indicating that the compound contained extra ring. The signals of cyclic methylene, methine and oxygenated carbon suggested the presence of a pyrane moiety. Furthermore, the compound showed similar ^1^H-NMR spectra to that of 8-benzoyl-3,5,7-trihydroxy-2,2-dimethoxychroman isolated from *Leontonyx spathulatus* [17]. The only difference is the nature of the side chain at C-8, which showed typical signals of 2-methyl-1-propanone (Table 1 and Table 2). The HMBC confirmed the pyrane ring connectivity and the side chain at C-8 and showed correlations between H-4/C-9, C-5, C-10, C-2; H-6/C-5, C-7, C-10, C-1 and H-2ʹ/C-1ʹ and C-8. Other 2D spectra thereby supported the structure of **3**. The absolute configuration at C-3 could not be ascertained directly from the data obtained however, the comparison of the measured specific rotation value (${\text{[α]}}_{D}^{20}$ +1.8, (*c* 0.04 MeOH)] of **3** with the reported value [reported ${\text{[α]}}_{D}^{20}$ −1.2 (c 0.029, MeOH) of similar structure containing “identical stereo centre” and similar coupling constant values of H-3 and H-4 [19] indicated 3*S* isomer of **3**. Accordingly, the structure of **3** was established as 8-(2-methyl-1-propanone)-3*S*,5,7-trihydroxyl-2,2-dimethylchromane (helinivene C).

**Table 1:** ^1^H-NMR (400 MHz: *m*, *J* Hz) spectral data of compounds **1**–**10** in CDCl_3_ (**4**, **5**, **7** and **10**) or CD_3_COCD_3_ (**1**–**3**, **6**, **8**, **9**).

	1	2	3	4	5	6	7	8	9	10
1	-	-	-	-	-	-	-	-	-	-
2	-	-	-	-	-	-	-	-	-	-
3	-	-	3.81 *br* t 6.8	5.91 *s*	5.91 *s*	-	-	-	-	-
4	-	-	2.87 *dd* (5.5, 16.6); 2.51 *dd* (7.7, 16.6)	-	-	-	-	-	-	-
5	6.07 *s*	6.03 *s*	-	5.91 *s*	5.91 *s*	6.05 *s*	5.83 *s*	-	6.06 *s*	5.88 *s*
6	-	-	5.99 *s*	-	-	-	-	-	-	-
7	3.38 *d*, 7.5	3.02 *dd*, 15.8, 2.0 2.77 *dd*, 15.8, 7.8	-	-	-	3.22 *d*, 6.5	3.32 *d*, 7.0	3.45 *d*, 7.6	3.23 *d*, 6.8	3.42 *d*, 7.0
8	5.54 *t*, 7.5	4.34 *br* *d*, 7.8	-	3.70 *sext*, 6.4	3.83 *sept*, 6.4	5.20 *t*, 6.5	5.21 *t*, 7.0	5.51 *t*, 7.6	5.21 *t*, 6.8	5.38 *t*, 7.0
9	-	-	-	1.38 *m*	1.59 *d*, 6.4	-	-	-	-	-
10	4.82 *s*	4.97 *s*, 4.79 *s*	-	1.14 *d,* 6.4	1.59 *d*, 6.4	1.61 *s*	1.73 *s*	4.82 *s*	1.73 *s*	4.73 *s*
11	1.71 *s*	1.81 *s*	1.37 *s*	0.89 *t,* 7.6	-	1.72 *s*	1.78 *s*	1.71 *	1.62 *s*	1.70 *s*
12	-	-	1.45 *s*	-	-	-	-	-	-	-
1ʹ	-	-	-	4.47 *d*, 6.7	4.47 *d*, 6.7	-	-	-	-	-
2ʹ	-	-	3.92 *sept* 6.7	5.42 *t,* 6.7	5.42 *t*, 6.7	3.04 *t*, 7.4	3.74 *sext*, 6.8	3.08 *t*, 7.2	3.98 *sept*, 6.8	3.89 *sept*, 6.8
3ʹ	7.59 *m*	7.49 *br*. *d*, 7.2	1.15 *d* 6.7	-	-	1.67 *sext*, 7.4	1.37 *m*	1.71 *	1.12 *s*	1.43 *s*
4ʹ	7.38 *m*	7.40 *br*. *t*, 7.6	1.15 *d* 6.7	1.77 *s*	1.77 *s*	0.94 *t*, 7.4	0.88 *t*, 7.2	0.99 *t*, 7.2	1.12 *s*	1.43 *s*
5ʹ	7.47 *m*	7.45 *br* *t*, 7.6		1.71 *s*	1.71 *s*	-	1.13 *d*, 6.8	-	-	-
6ʹ	7.38 *m*	7.40 *br*. *t*, 7.6	-	-	-	-	-	-	-	-
7ʹ	7.59 *m*	7.49 *br*. *d*, 7.2	-	-	-	-	-	-	-	-
COCH_3_	2.02 s	-	-	-	-	-	-	2.07 *s*	-	2.05 *s*
2-OH	12.27 *s*	-	-	-	9.92 *s*	14.07 *s*	11.76 *s*	14.0 *s*	14.09 *s*	12.88 *s*
4-OH	9.28 *s*	-	-	-	-	9.31 *br s*	8.81 *br s*	9.66 *br s*	9.33 *br s*	8.23 *br* *s*
6-OH	8.99 *s*	-	-	-	9.92 *s*	-	-	9.25 *br s*	-	-

* overlapped signals.

**Table 2:** ^13^C-NMR spectral data of compounds **1**–**10** in CDCl_3_ (**4**, **5**, **7** and **10**) or CD_3_COCD_3_ (**1**–**3**, **6**, **8**, **9**).

.	1	2	3	4	5	6	7	8	9	10
1	104.5 *s*	104.9 *s*	-	104.5 *s*	104.0 *s*	105.1 *s*	105.7 *s*	105.4 *s*	104.3 *s*	104.0 *s*
2	163.4 *s*	163.2 *s*	27.1 *s*	164.6 *s*	164.7 *s*	165.2 *s*	162.6 *s*	165.4 *s*	165.2 *s*	163.5 *s*
3	106.4 *s*	106.5 *s*	80.0 *d*	95.1 *d*	95.1 *d*	107.9 *s*	104.7 *s*	107.0 *s*	108.0 *s*	105.8 *s*
4	163.2 *s*	161.2 *s*	69.2 *t*	164.6 *s*	164.7 *s*	160.5 *s*	159.9 *s*	162.8 *s*	162.5 *s*	160.7 *s*
5	96.0 *d*	96.6 *d*	157.2 *s*	95.1 *d*	95.1 *d*	95.0 *d*	95.4 *d*	95.3 *d*	95.1 *d*	95.3 *d*
6	160.0 *s*	160.1 *s*	163.4 *s*	164.6 *s*	164.7 *s*	162.6 *s*	160.7 *s*	161.1 *s*	160.1 *s*	159.4 *s*
7	21.91 *t*	28.9 *t*	167.0 *s*	210.1 *s*	210.2 *s*	22.0 *t*	21.6 *t*	22.1 *t*	22.1 *t*	21.0 *t*
8	129.2 *d*	77.7 *d*	105.6 *s*	45.9 *d*	39.3 *d*	124.3 *d*	121.6 *d*	129.6 *d*	124.2 *d*	128.9 *d*
9	130.4 *s*	148.8 *s*	96.6 *s*	26.9 *t*	19.2 *q*	130.8 *s*	135.9 *s*	130.4 *s*	130.8 *s*	129.9 *s*
10	63.7 *t*	110.8 *t*	100.6 *s*	11.9 *q*	19.2 *q*	26.0 *q*	25.8 *q*	64.0 *t*	17.9 *q*	64.1 *t*
11	21.6 *q*	19.0 *q*	20.3 *q*	16.6 *q*	-	17.9 *q*	17.9 *q*	21.8 *q*	25.9 *q*	21.1 *q*
12	-	-	20.2 *q*	-	-	-	-	-	-	-
1ʹ	200.3 *s*	200.1 *s*	211.1 *s*	65.1 *t*	65.1 *t*	206.5 *s*	210.8 *s*	206.9 *s*	210.9 *s*	210.8 *s*
2ʹ	143.0 *s*	143.4 *s*	40.3 *d*	118.7 *d*	118.7 *d*	46.5 *t*	45.9 *d*	46.8 *t*	39.7 *d*	39.2 *d*
3ʹ	128.4 *d*	128.9 *d*	26.1 *q*	139.2 *s*	139.2 *s*	19.0 *t*	26.9 *t*	19.2 *t*	19.8 *q*	19.3 *q*
4ʹ	129.1 *d*	129.6 *d*	21.1 *q*	18.2 *q*	18.2 *q*	14.4 *q*	11.9 *q*	14.6 *q*	19.8 *q*	19.3 *q*
5ʹ	131.6 *d*	132.4 *d*	-	25.8 *q*	25.8 *q*	-	16.9 *q*	-	-	-
6ʹ	129.1 *d*	129.6 *d*	-	-	-	-	-	-	-	-
7ʹ	128.4 *d*	128.9 *d*	-	-	-	-	-	-	-	-
CO	171.3 *s*	-	-	-	-	-	-	171.5 *s*	--	172.6 *s*
CH_3_	20.9 *q*	-	-	-	-	-	-	21.1 *q*	-	21.2 *q*

Compounds **4** and **5** showed very similar NMR spectra. ^1^H-NMR showed signals of two aromatic protons at δ_H_ 5.91 s, and a typical signal of *O*-prenyl group. The only difference between **4** and **5** is the side chain at C-1; in the case of **4**, ^1^H-NMR showed a typical 2-methylbutanone, while **5** showed 2-methylpropanone. **4** was identified as 1-(2-methylbutanone)-4-*O*-prenyl-phloroglucinol and **5** as 1-(2-methylpropanone)-4-*O*-prennyl-phloroglucinol. Both **4** and **5** were previously isolated from *H*. *crispum* [20]. **6** showed a typical NMR signals like that of 1-(butanone)-3-prenyl-phloroglucinol [21], while **7** identified as 1-(2-methylbutanone)-3-prenyl-phloroglucinol. Compounds **6** and **7** were isolated previously from *H*. *gymnoconum* [22]. Compound **8** was identified as 1-butanone-3-(3-methylbut-2-enylacetate)-phloroglucinol [21], **9** was identified as 1-(2-methylpropanone)-3-prenylphloroglucinol and previously isolated from *H*. *mimetes* [23] and **10** identified as caespitate, was first isolated from *H*. *caespititium* [23].

Compound **11** was obtained as a white solid and is the only alcohol triterpene detected in this study. **11** was identified as 3β-24-dihydroxyterexer-14-ene and reported previously from *Erythroxylum* *passerium* [24]. According to SciFinder and the dictionary of natural products, the occurrence of **11** from *Helichrysum* species is reported for the first time.

### 2.1. Total Antioxidant Capacities of Acylphloroglucinols

The greater the number of hydroxyl groups in phenolic compounds, the higher is the antioxidant activity. Hydroxyl radicals are an extremely reactive oxygen species, capable of modifying almost every molecule in the living cell. Moreover, hydroxyl radicals are capable of quick initiation of the lipid peroxidation process by abstracting hydrogen atoms from unsaturated fatty acids [25].

The total antioxidant capacities of a methanolic extract (HF) and its isolated compounds (**1**–**11**) were evaluated using a battery of assays which included the FRAP, ORAC, TEAC and Fe^2+^-induced microsomal lipid peroxidation assays. Helinivenes A and B (**1** and **2**) when compared to EGCG, displayed significantly higher ORAC_OH_ values (64.85 ± 10.95; 94.97 ± 5.88 *vs.* 3.91 ± 4.65) × 10^6^ µM TE/g, respectively. The same trend of significantly increased capacity for **1** and **2** than EGCG when using the peroxyl radical absorbance capacity assay was observed (22,671.78 ± 26.72; 45,095.82 ± 31.99 *vs*. 14,693.0 ± 5.53) µM TE/g respectively, while compounds **6**–**10** showed a mild peroxyl absorbance capacity (range 13,544–16,735 µM TE/g), and hydroxyl absorbance capacity (range 16.26–24.24) × 10^6^ µM TE/g, respectively, as shown in Table 3.

The findings corroborate that of previous reports on the structure-activity relationship of antioxidant activities of monomeric phenolic compounds, prenylated coumarins and phloroglucinol derivatives, where their respective antioxidant activities were found to depend on the degree of hydroxylation and extent of conjugation [25,26,27]. The potential antioxidant activities of **1** and **2**, as well as moderate antioxidant activities demonstrated by **6**–**10** may be attributed to the presence of hydroxyl groups, which act as good hydrogen donors, thereby forming phenolate ions as intermediates. These intermediates are stabilized by resonance when unpaired electrons at the *m*-positions of the aromatic ring are delocalized [26]. To further elucidate the mechanism of action of the isolated compounds, we established whether the isolated compounds have the ability of transferring electrons to free radicals by measuring their FRAP and TEAC activities at different pH. The results in Table 3 shows, as expected, that phloroglucinols containing aromatic acyl groups (**1** and **2**) possessed higher antioxidant (when measured as FRAP, TEAC) activities relative to their monocyclic (**4**–**10**), and bicyclic (**3**) counterparts with aliphatic acyl substitutions. The observed tendencies demonstrated by these compounds are in accordance with data previously reported in the literature [27,28]. It is evident therefore that the isolated compounds **1** and **2** possessed interesting antioxidant activities, expressed as their trolox (TEAC, ORAC) and ascorbic acid (FRAP) equivalents.

**Table 3:** Total antioxidant capacities of a methanolic extract of *H. niveum* and its constituents.

Sample	Peroxyl	Hydroxyl × 10^6^ µMTE/g *	Prooxidant	TEAC	FRAP µMAAE/g *	Anti-Lipid peroxidation IC_50_; (µg/mL) **
HF ***	4553.13 ± 17.77	53.77 ± 8.42	17.29 ± 0.99	1449.54 ± 3.09	437.64 ± 6.86	27.73 ± 4.00
**1**	22671.78 ± 26.72	64.85 ± 10.95	8.92 ± 1.15	19545.00 ± 10.25	2530.54 ± 0.92	5.12 ± 0.90
**2**	45095.82 ± 31.99	94.97 ± 5.88	756.90 ± 1.98	43615.73 ± 6.66	4950.08 ± 0.65	3.55 ± 1.92
**3**	3937.78 ± 25.85	5.99 ± 4.52	41.73 ± 8.20	6001.40 ± 5.63	155.65 ± 12.02	>50.00
**4**	5406.65 ± 1.39	3.92 ± 14.47	7.02 ± 4.02	1629.10 ± 2.03	194.27 ± 1.78	44.62 ± 2.08
**5**	6053.87 ± 18.67	5.60 ± 0.79	12.33 ± 6.62	2673.62 ± 1.68	446.64 ± 8.07	20.22 ± 2.72
**6**	13544.93 ± 12.13	18.07± 3.47	64.37 ± 0.22	4316.61 ± 1.06	1183.52 ± 6.20	30.80 ± 2.12
**7**	14139.74 ± 5.96	23.12 ± 18.94	73.87 ± 1.10	9998.71 ± 2.66	1029.03 ± 0.66	27.49 ± 3.19
**8**	14218.17 ± 12.36	16.70 ± 1.25	76.59 ± 0.27	6757.40 ± 4.69	1203.02 ± 2.07	27.56 ± 0.63
**9**	13639.27 ± 17.62	24.24 ± 1.71	69.54 ± 0.29	4423.32 ± 3.11	1096.01 ± 1.12	20.95 ± 2.62
**10**	16735.55 ± 21.72	16.26 ± 15.39	92.31 ± 1.59	8705.14 ± 1.83	1019.28 ± 1.79	20.50 ± 4.01
**11**	1193.10 ± 1.68	0.89 ± 7.34	31.38 ± 7.00	1323.61 ± 1.76	197.99 ± 3.90	>50.00
EGCG	14970.09 ± 5.53	3.91 ± 4.65	6.48 ± 1.19	11545.44 ± 17.28	3326.45 ± 5.76	1.04 ± 1.02

* Data expressed as ± % STDEV; ** Data are given as IC_50_ ± STDEV with tested samples screened at 50.00 μg/mL; HF ***: methanolic extract of *H. niveum*.

Over production of ROS results in an attack of not only DNA, but also other important cellular components, including the polyunsaturated fatty acids (PUFAs) in cell membranes, which are highly susceptible to free-mediated oxidation [29]. Compounds **1** and **2** demonstrated a potent inhibitory activity against the Fe^2+^-induced lipid peroxidation (IC_50_ = 5.12 ± 0.90; 3.55 ± 1.92) µg/mL, respectively (Table 3) in a competitive manner to that of EGCG (IC_50_ 1.04 ± 1.02) µg/mL. This result is in agreement with previous work, where phloroglucinol were found to possess similar Fe^2+^-induced inhibition against lipid peroxidation to that of BHA and α-tocopherol [30]. Our observations further explain how the poly-hydroxylated ring A of **1** and **2** can be an active “anti-lipid peroxidation skeleton”. However, it is possible that the second aromatic ring in **1** and **2** can enhance the contact of these antioxidants with lipids, and consequently resulting in an efficient termination of the chain reaction [31].

### 2.2. The Skin Aging Related-Enzymes Inhibitory Activities of Acylphloroglucinols

The methanolic extract (HF) and the isolated compounds **1**–**11** were evaluated for their anti-tyrosinase activity at the optimum concentration of 100 µg/mL. Moderate inhibition of tyrosinase activity demonstrated by these compounds (specifically **1** and **2**, as indicated in Table 4), might depend on the substitution pattern of the hydroxyl groups and non-existence of the catechol group, which may form hydrogen bonds to a chelating site of the enzyme [32,33].

Further attempts were made to correlate the chemistry of phenol-metal chelation between Fe^2+^ (in lipid peroxidation assay) to that of Cu^2+^ (in tyrosinase assay), but the anti-tyrosinase result indicated that kojic acid is eight times more active than **1** and **2**. We therefore proposed that the mechanism of Fe^2+^ chelation in the lipid peroxidation assay may be totally different from that of Cu^2+^ chelation in the tyrosinase assay, despite both metal ions (Fe^2+^ and Cu^2+^) being bivalent in nature. The results obtained in Table 4 demonstrated a less potent inhibition against elastase (with IC_50_ ranged 25.31–69.61 µg/mL), in comparison to oleanolic acid (IC_50_ 10.08 ± 8.47 µg/mL). No scientific report has pointed out the reason for such activity, except previous data indicating a less potent activity of the non-prenylated acylphloroglucinol against inhibition of the release of leukocyte elastase [34]. None of the compounds demonstrated activity against acetylcholinesterase except at a high concentration of 250.00 µg/mL, possibly due to the absence of nitrogen atom-containing compounds in our isolated products [35].

**Table 4:** Enzyme inhibitory effect of a methanolic extract of *H. niveum* and its constituents.

Sample	Inhibitory Activities (IC_50_ ± % STDEV; µg/mL) *		
	Tyrosinase	Elastase	Acetylcholinesterase
HF **	76.02 ± 4.91	92.97 ± 8.90	257.98 ± 4.01
**1**	35.63 ± 4.67	>100.00	267.96 ± 3.88
**2**	26.72 ± 5.05	>100.00	267.96 ± 1.15
**3**	80.31 ± 3.31	25.31 ± 7.85	272.95 ± 4.61
**4**	>100.00	45.63 ± 15.27	272.95 ± 1.67
**5**	>100.00	69.61 ± 4.71	252.99 ± 2.41
**6**	>100.00	35.47 ± 7.85	252.99 ± 2.13
**7**	>100.00	66.72 ± 5.21	260.48 ± 5.04
**8**	>100.00	63.44 ± 7.56	248.00 ± 5.41
**9**	>100.00	61.563 ± 4.69	250 50 ± 2.49
**10**	>100.00	58.75 ± 5.42	250.50 ± 2.21
**11**	>100.00	>100.00	313.19 ± 4.58
Kojic acid	3.51 ± 6.01	-	-
Oleanolic acid	-	10.08 ± 8.47	-
Galanthamine	-	-	10.98 ± 1.03

* Data are given as IC50 ±STDEV with tested samples screened at 100.00 μg/mL for anti-tyrosinase anti-elastase assays, while 500.00 μg/mL was considered as upper concentration limits for acetylcholinesterase assay. HF **: Methanolic extract of *H. niveum*.

## 3. Experiment Section

### 3.1. Chemicals and Reagents

The organic solvents methanol, acetonitrile (HPLC graded), ethanol, ethyl acetate, dichloromethane, hexane (technical grade), deuterated chloroform and acetone were supplied by Merck (Cape Town, South Africa). Sulfuric acid and acetic acid were secured from Kimix (Cape Town, South Africa). Kojic acid, galanthamine, oleanolic acid, epigallocatechin gallate (EGCG), trolox (6-Hydroxyl-2,5,7,8-tetramethylchroman-2-carboxylic acid), ABTS (2,2ʹ-Azino-bis (3-ethylbenzo thiazoline-6-sulfonic acid) diammonium salt), 5,5ʹ-dithiobis-(2-nitrobenzoic acid (DTNB), acetythiocholine iodide (ACTI), potassium peroxodisulfate, fluorescein sodium salt, AAPH (2,2ʹ-Azobis (2-methylpropionamidine) dihydrochloride, perchloric acid, TPTZ (2,4,6-tri[2-pyridyl]-*S*-triazine, Iron (III) chloride hexahydrate, tris-HCl, sepharose (wet bead diameter, 60–200 µm), copper sulfate, hydrogen peroxide, and *N*-succ-(Ala)3-nitroanilide (SANA) were secured from Sigma-Aldrich, Inc. (St. Louis, MO, USA).

### 3.2. Plant Material

The aerial parts of the plant were collected in October 2012, from Jonkershoek (about 9 km SE Stellenbosch) nature reserve, Western Cape, South Africa. The plant was kindly identified by Dr. Christopher Cupido (SANBI, Kirstenbosch). A voucher specimen (Herbarium number NBG1458801) was deposited at the Compton Herbarium, South African National Biodiversity Institute (SANBI), Kirstenbosch, South Africa.

### 3.3. Apparatus

**Preparative HPLC**: Preparative HPLC of sample purification was performed on an Agilent Technologies (Santa Clara, CA, USA) 1200 series, equipped with UV detector, manual injector, quaternary pump, vacuum degasser, column compartment (all experiments were done at room temperature) and reversed phase C18 column SUPELCO (25 × 1.0 cm). The flow rate was set at 1.5 mL/min and detection wavelength at 254 nm.

**Open-Column Liquid Chromatography and Thin-Layer Chromatography**: Liquid chromatography (CC) was performed over silica gel 60 (0.040−0.063 mm particle size, Merck, Cape Town, South Africa) and sephadex LH-20 (Sigma-Aldrich, Cape Town, South Africa) as stationary phases, supported with glass column of different diameters. Pre-coated plates of silica gel 60 F_254_ (Merck, Germany) were used for TLC analysis. Chemical profiles of the fractions were identified based on the color produced after viewing under UV and then spraying with the detecting reagent (vanillin/sulfuric acid) [36].

**Optical Rotation**: Optical activity measurements were conducted in methanol using a Autopol III Polarimeter (Rudolph research analytical, Hackettstown, MA, USA).

**The NMR Analyses**: ^1^H- and ^13^C-NMR spectra were recorded at 25 °C, using deuterated chloroform or acetone on a Bruker Avance 400 MHz NMR spectrometer (Bruker, Rheinstetten, Germany). Chemical shifts of ^1^H (δ_H_) and ^13^C (δ_C_) were reported in parts per million (ppm) relative to internal reference (CDCl_3_; 7.24/77.0; CD_3_COCD_3_; 2.05/206.68).

***In Vitro* Biological Activity Measurements**: All antioxidant assays including ferric-ion reducing antioxidant power (FRAP), trolox equivalent absorbance capacity (TEAC), lipid peroxidation, and enzyme inhibition (tyrosinase, elastase and acetylcholineesterase) assays were measured using a Multiskan spectrum plate reader, while the automated ORAC assay was read on a Fluoroskan spectrum plate reader. Greiner^®^ F Bottom (white and black) 96-well micro-plates (Greiner Bio-One GmbH, Frickenhausen, Germany) were used for all biological assays of antioxidant and enzymes inhibitory activity.

### 3.4. Preparation of Crude Extract

The aerial parts (stem, leave and flower; 400 g) were air dried at room temperature, blended and extracted with methanol (2.5 L × 2) at room temperature (25 °C) for 48 h. The methanol extract was evaporated till dryness under reduced pressure at 40 °C to yield 15.0 g (3.75%).

### 3.5. Fractionation and Isolation of Constituents Using Semi-Prep HPLC

The total extract (15 g) was applied to a silica gel column and eluted using a gradient of hexane:ethyl acetate: methanol in the order of increasing polarity which resulted in 48 fractions (500 mL each). Fractions collected were combined according to their thin layer chromatographic (TLC) profiles to yield seventeen main fractions labeled I–XVII.

The main fraction XI (1.6 g) was chromatographed on a silica gel column using a gradient of hexane:ethyl acetate (1:0–1:1) (*v*/*v*). Sub fraction XI-6 (340 mg) was re-chromatographed on Sephadex using 5% aq. ethanol and sub-fractions obtained were injected into the HPLC using a gradient of acetonitrile and de-ionized water (60:40 to 80% acetonitrile in 30 min, then 100% for 10 min) producing three compounds **1** (R_t_ 25 min, 29 mg, 0.0073%), **5** (R_t_ 34 min, 31 mg, 0.0075%) and **6** (R_t_ 37 min, 29 mg, 0.0073%). Sub fraction XI-7 (180 mg) was chromatographed on Sephadex using 5% aqueous ethanol, with HPLC (75:25 to 90% acetonitrile in 30 min, then 100% for 10 min) producing **3** (R_t_ 17 min, 44 mg, 0.011%). Main fraction IX (305 mg) was chromatographed on Sephadex using 5% aqeuous ethanol, then injected into the HPLC (60:40 to 80% acetonitrile in 30 min, then 100% for 10 min) producing **4** (R_t_ 35 min, 23 mg, 0.0058%). Main fraction VII (2.6 g) was chromatographed on silica gel column using a gradient elution of hexane: ethyl acetate 100:0 to 7:3 (*v*/*v*). Sub fractions VII-5 (72 mg) was injected to the HPLC (70:30 to 90% acetonitrile in 20 min, then 100% for 15 min) producing two compounds as **7** (R_t_ 22 min, 17 mg, 0.0043%), and **9** (R_t_ 25 min, 24 mg, 0.006%). Main fraction VI (200 mg) was chromatographed on sephadex using 5% aqueous ethanol, then injected into the HPLC (80:20 to 90% acetonitrile in 20 min, then 100% for 15 min) producing compound **8** (R_t_ 23 min, 21 mg, 0.0053%). Main fraction III (130 mg) was chromatographed on Sephadex using 5% aqueous ethanol, then injected into the HPLC (75:25 to 90% acetonitrile in 30 min, then 100% for 10 min) producing two compounds as **10** (R_t_ 22 min, 13 mg, 0.0033%), and **2** (R_t_ 28 min, 15 mg, 0.0038%). Main fraction IV (155 mg) was chromatographed on a Sephadex column using 5% aqueous ethanol producing compound **11** (53 mg, 0.0133%).

### 3.6. Structural Identification of Compounds

All compounds were identified based on extensive spectroscopic data analyses, including one-dimensional (^1^H- and ^13^C-) and two-dimensional NMR (HSQC, HMBC and NOESY).

### 3.7. Total Antioxidant Capacity Assays

**Ferric-Ion Reducing Antioxidant Power (FRAP) Assay**: Working FRAP reagent was prepared in accordance to the method described previously [37]. Absorbance was measured at 593 nm. l-ascorbic acid was used as a standard and the results were expressed as μM ascorbic acid equivalents per milligram dry weight (μM AAE/g DW) of the test samples.

**Automated Oxygen Radicals Absorbance Capacity (ORAC) Assay**: ORAC was measured according to the method previously described [16]. The method measures the antioxidant scavenging capacity for (a) peroxyl radicals generated by thermal decomposition of 2,2ʹ-azobis (2-aminopropane) dihydrochloride (AAPH; ORAC_ROO_ assay); (b) hydroxyl radical (ORAC_OH_ assay), generated by H_2_O_2_-Cu^2+^ (H_2_O_2_, 0.3%; Cu^2+^ [as CuSO_4_], 18 μM, or (c) Cu^2+^ [as CuSO_4_], 18 μM as a transition metal oxidant at 37 °C. ORAC values were expressed as micromoles of trolox equivalents (TE) per milligram of test sample, except when Cu^2+^ (without H_2_O_2_) was used as an oxidant in the assay. In the presence of Cu^2+^ without H_2_O_2_, test samples acted as prooxidants rather than antioxidants in the ORAC assay. The copper-initiated prooxidant activity was calculated using [(Area_Blank_ − Area_Sample_)/Area_Blank_] × 100 and expressed as prooxidant units; one unit equals the prooxidant activity that reduces the area under the fluorescein decay curve by 1% in the ORAC assay.

**Trolox Equivalent Absorbance Capacity (TEAC) Assay**: The total antioxidant capacity of the test samples in terms of TEAC were measured using a previously described method [38]. Absorbance was read at 734 nm at 25 °C in a plate reader and the results were expressed as μM trolox equivalents per milligram dry weight (μM TE/g DW) of the test samples.

**Inhibition of Fe (II)-Induced Microsomal Lipid Peroxidation Assay**: The thiobarbituric acid reactive substances (TBARs) method was used to evaluate the inhibition of lipid peroxidation as previously described [39] with minor adjustment. Rat liver microsomes were isolated from an S9 fraction using a Sepharose column with 0.01 M potassium phosphate buffer; pH 7.4, supplemented with 1.15% KCl at 5 °C. Absorbance was measured at 532 nm and the percentage inhibition of TBARs formation relative to the positive control was recorded.

### 3.8. Enzyme Inhibition Assays

**Tyrosinase Enzyme Assay:** The tyrosinase inhibitory activity was measured using the spectrophotometric method previously described [16]. Samples were dissolved in DMSO to a stock solution of 1 mg/mL, and further dilutions were then done with 50 mM sodium phosphate buffer (pH 6.5) for all working solutions. Kojic acid was used as control drug. In the wells of a 96-well plate, 70 µL of each sample working solution was combined with 30 μL of tyrosinase (500 Units/mL in sodium phosphate buffer) in triplicate. After incubation at room temperature for 5 min, 110 μL of substrate (2 mM l-Tyrosine) was added to each well. Final concentrations of the crude extract, isolated compounds, and positive control ranged from 1.0–100 μg/mL. Incubation commenced for 30 min at room temperature and the enzyme activity was determined by measuring the absorbance at 490 nm. The percentage of tyrosinase inhibition was calculated as follows:

Tyrosinase inhibition (%) = [(A − B) − (C − D)]/(A − B) × 100
(1)

where A is the absorbance of the control with the enzyme, B is the absorbance of the control without the enzyme, C is the absorbance of the test sample with the enzyme and D is the absorbance of the test sample without the enzyme.

**Elastase Inhibition Assay**: Inhibition of elastase by the test samples was assayed using *N*-succyl-(Ala)-3-nitroanilide (SANA) as the substrate, monitoring the release of *p*-nitroanilide by the method described previously [16]. The inhibitory activity determined the intensity of color released during cleavage of SANA by the action of elastase. Briefly, 1 mM SANA was prepared in 0.1 M Tris-HCl buffer pH 8.0 and 200 μL of this solution was added to the 20 μL of sample solution in a 96-Well plate. The mixtures were vortexed and preincubated for 10 min at 25 °C and then 20 μL of elastase from porcine pancrease (0.03 Units/mL) was added. The mixtures were further incubated for 10 min and the absorbance was measured at 410 nm. Methanol was used as control, while oleanolic acid used as a positive control. The percentage of elastase inhibition was calculated as follows:

Elastase inhibition (%) = (1 − B/A) × 100
(2)

where A is the enzyme activity without sample and B is the activity in the presence of the sample.

**Acetylcholinesterase Inhibition Assay**: Acetylcholinesterase (AChE) inhibitory activity was measured using Ellman’s method as previously described [40] with minor modifications. AChE catalysis the hydrolysis of acetythiocholine iodide (ACTI as substrate) to thiocholine, which can then react with Ellman’s reagent (DTNB) to produce 5-thio-2-nitrobenzoate (yellow color). In the presence of samples (inhibitors), the release of 5-thio-2-nitrobenzoate (yellow color) is reduced and it is monitored by measuring the absorbance at 450 nm.

In a 96-Well plate, the reaction mixture contains 50 µL of samples pre-incubated with 125 µL of 3 mM 5,5ʹ-Dithiobis-(2-nitrobenzoic acid), and 25 µL AChE (2.0 U/mL), in 50 mM tris-HCl buffer (pH 8.0), with 0.1% bovine serum albumin (BSA) for 15 minutes at 25 °C. Subsequently, 25 µL (15 mM) ACTI was added to the incubation mixture and further incubated for 10 min at 25 °C. Sample control was prepared by adding sample solution to all reagents without AChE, blank contained tris-HCl in the presence of AChE, while galanthamine was used as reference. Absorbance was recorded at 450 nm. The percent AChE inhibitory activity is given by:

[A_0_ − (B − C)]/A_0_ × 100 (3)

where A_0_ = full enzymatic reaction; B = Activity in the presence of sample; and C = Activity without sample.

## 4. Conclusions

Fractionation of a methanol extract (HF) was carried out using standard chromatographic methods. A total of eleven pure compounds (**1**–**11**) were isolated, of which three were newly reported. Their chemical structures were established by spectroscopic methods. Several reports have convincingly shown a close relationship between antioxidant activity and the structural activity of phenolic compounds. On the basis of our findings, the prerequisite for a strong antioxidant activity assessed in these assays was the presence of an aromatic acyl-substituent, which may be valuable as a potential antioxidant and lipid peroxidation preventer, in the absence of prooxidant behavior. However, we had fully established the mode of actions through which the isolated compounds behave as a good source of antioxidant and moderate anti-tyrosinase and anti-elastase activities demonstrated by the *Helichrysum niveum* constituents. This study is the first report on *in vitro* antioxidant and aging-related enzyme inhibitory activity of a methanol extract of *H. niveum* and its constituents. The total antioxidant capacities and aging-related enzyme inhibitory activities demonstrated can be considered significant and informative as a guide for further studies.
